# Synthesis and characterization of enanti­opure planar–chiral phospho­rus-linked diferrocenes

**DOI:** 10.1107/S2053229621001996

**Published:** 2021-02-25

**Authors:** Philipp Honegger, Alexander Roller, Michael Widhalm

**Affiliations:** a Department of Computational Biological Chemistry, University of Vienna, Waehringer Strasse 17, A-1090 Vienna, Austria; b Department of Inorganic Chemistry, Faculty of Chemistry, University of Vienna, Waehringer Strasse 42, A-1090 Vienna, Austria; c Department of Chemical Catalysis, Faculty of Chemistry, University of Vienna, Waehringer Strasse 38, A-1090 Vienna, Austria

**Keywords:** crystal structure, metal organic, three-dimensional structure, ferrocene, phosphine sulfide, chirality, planar chirality

## Abstract

In the course of an ongoing synthetic project on cyclic diferrocenylphosphines, we obtained a group of planar–chiral diferrocenyl com­pounds useful as precursors for subsequent cyclization. Here we report the crystal structures of two sym­metric com­pounds [(Fc^A^)_2_(Ph)P], one of which contains four stereogenic centres (two C chiral and two planar chiral centres), *i.e.* 1,1′-(phenyl­phos­phane­di­yl)bis­{(2*S*_p_)-2-[(1*R*)-1-(acet­yloxy)eth­yl]ferrocene}, [Fe_2_(C_5_H_5_)_2_(C_24_H_25_O_4_P)], and the other phosphine sulfide is a purely planar–chiral com­pound (two planar chiral centres), *i.e.* bis[(2*S*_p_)--2-ethenylferrocen-1-yl]phenylphosphane sulfide, [Fe_2_(C_5_H_5_)_2_(C_20_H_17_PS)]. Owing to the stereocentres present, reactions performed on [(Fc^A^)_2_(Ph)P]-type com­pounds strongly favour one ferrocene unit over the other due to diastereoselectivity. Furthermore, we present four related structures where the ferrocene units are not identical [(Fc^A^)(Fc^B^)(Ph)P]. These are {(2*S*_p_)-2-[(1*R*)-1-(acet­yloxy)eth­yl]ferrocen-1-yl}[(2*S*_p_)-2-ethenylferrocen-1-yl]phenyl-(*S*)-phosphine sulfide, [Fe_2_(C_5_H_5_)_2_(C_22_H_21_O_2_PS)], [(2*S*_p_)-2-ethenylferrocen-1-yl]{(2*S*_p_)-2-[(1*R*)-1-hy­droxy­eth­yl]ferrocen-1-yl}phenyl-(*S*)-phosphine sulfide, [Fe_2_(C_5_H_5_)_2_(C_20_H_19_OPS)], {(2*S*_p_)-2-[(1*R*)-1-(acet­yloxy)eth­yl]ferro­cen-1-yl}{(2*S*_p_)-2-[(1*R*)-1-hy­droxy­eth­yl]ferrocen-1-yl}phenyl-(*R*)-phos­phine sulfide, [Fe_2_(C_5_H_5_)_2_(C_22_H_23_O_3_PS)], and {(2*S*_p_)-2-[(1*R*)-1-benzyl­amino)­eth­yl]ferrocen-1-yl}[(2*S*_p_)-2-ethenylferrocen-1-yl]phenyl-(*S*)-phosphine sulfide, [Fe_2_(C_5_H_5_)_2_(C_27_H_26_NPS)]. All of the structures are accessible in one step from known precursors.

## Abstract 2

Six new homochiral diferrocenyl derivatives have been synthesized, one of which is purely planar–chiral. Even if the two diferrocene subunits are identical, they are distinguished due to their positions relative to the substituents at the phospho­rous prochiral centre.

## Introduction   

Metallocenes decorated with at least two different substituents on the same ring are planar–chiral (Schaarschmidt & Lang, 2013 ▸). They are useful as voluminous asymmetry-inducing groups in asymmetric transformations (Stepnicka, 2008 ▸). Even beyond academic research, disubstituted ferrocenes have been used in industrial asymmetric synthesis, for instance, in the hydrogenation of imines (Blaser *et al.*, 2007 ▸).

The potential of asymmetric induction may be improved by designing ligands including two planar–chiral ferrocene units. One example is the phospho­rous-linked diferrocene Pigiphos (Barbaro & Togni, 1995 ▸), developed by the group of Togni. A multitude of ligands for transition-metal com­plexes have been synthesized and applied in asymmetric homogeneous catalysis [*e.g.* Rh^I^ in hydro­silylations (Hayashi *et al.*, 1974 ▸), Rh^III^ in acetalizations (Barbaro *et al.*, 1999 ▸), Pd^II^ in hydro­aminations (Gischig & Togni, 2004 ▸, 2005 ▸), Ru^II^ in transfer hydrogenations (Barbaro *et al.*, 1997 ▸, 2003 ▸) and olefin cyclo­propanations (Lee *et al.*, 1999 ▸), and Ni^II^ in hydro­aminations (Fadini & Togni, 2004 ▸, 2007 ▸, 2008 ▸), hydro­phosphinations (Sadow & Togni, 2005 ▸), Naza­rov cyclizations (Walz & Togni, 2008 ▸) and dipolar cyclo­additons (Milosevic & Togni, 2013 ▸)].

The performance of catalysts may be enhanced further by making the angle of the two ferrocene units rigid *via* ring closure, yielding a diferrocenyl (macro)cycle (Xiao *et al.*, 2002 ▸), possibly with inclusion of another (planar–chiral) ferrocene unit (Wang *et al.*, 2006 ▸). Stanphos (Broggini, 2003 ▸) is a *P,P*-ligand diferroceno ring and has been employed in asymmetric hydro­alk­oxy­lations (Barreiro *et al.*, 2012 ▸) and the hy­droxy­lation of 1,3-ketoesters (Smith *et al.*, 2010 ▸).

**Figure 1:**
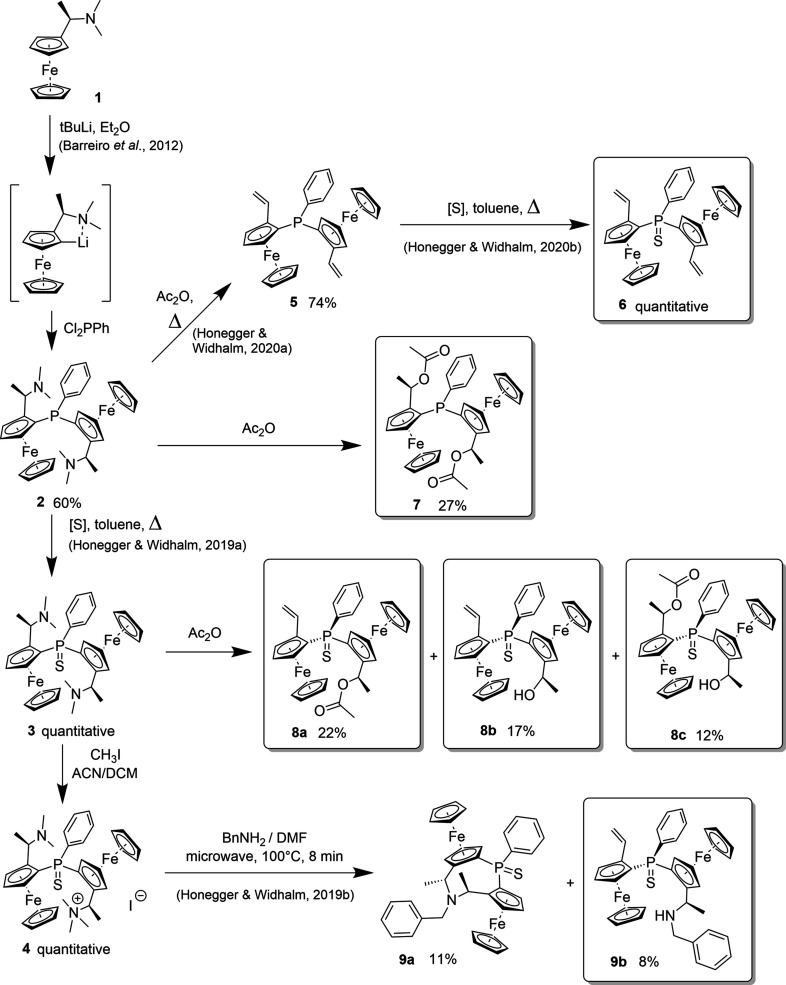
Synthetic route towards the crystallized phospho­rous-linked diferrocenes **6**, **7**, **8a**, **8b**, **8c** and **9b**.

In the course of the synthesis of cyclic diferrocene monophosphines with potential in asymmetric catalysis, we pre­pared com­pounds such as **6**–**9** (Fig. 1 ▸) potentially useful for a cyclization step. Their synthetic access starting from com­mercially available [1-(di­methyl­amino)­eth­yl]ferrocene (**1**) is outlined in Fig. 1 ▸. Beyond the desired cyclized products like **9a** (Honegger & Widhalm, 2019*b* ▸), several side products were isolated and characterized.

## Experimental   

### Synthesis and crystallization   

#### Synthesis of 1,1-(phenyl­phosphanedi­yl)bis­{(2*S*)-2-[(1*R*)-1-(acet­yloxy)eth­yl]ferrocene} (7)   

Di­amino­phosphine **2** (619 mg, 1.00 mmol; Barreiro *et al.*, 2012 ▸) was suspended in Ac_2_O (1 ml) in a flame-dried Schlenk tube under argon. The suspension was degassed and stirred for 7 d at room temperature, then for 7 h at 100 °C. From the dark-red solution, Ac_2_O was removed under reduced pressure and the residue was purified by column chromatography (SiO_2_, 0–100% EtOAc in hepta­ne) yielding di­acetate **7** (yield 177 mg, 27%) as pale-red crystals upon removal of the solvent (m.p. 155–156 °C). ^1^H NMR (400 MHz, CDCl_3_): δ 7.62–7.56 (*m*, 2H), 7.37–7.30 (*m*, 3H), 6.20 (*dt*, *J* = 2.5, 6.4 Hz, 1H), 5.99 (*dt*, *J* = 2.9, 6.4 Hz, 1H), 4.52 (*m*, 1H), 4.45 (*m*, 1H), 4.43 (*pt*, *J* = 2.5 Hz, 1H), 4.35 (*m*, 1H), 4.34 (*m*, 1H), 4.30 (*m*, 1H), 4.04 (*s*, 5H), 3.72 (*s*, 5H), 2.11 (*s*, 3H), 1.96 (*d*, *J* = 6.4 Hz, 3H), 1.56 (*d*, *J* = 6.4 Hz, 3H), 1.32 (*s*, 3H). ^31^P NMR: δ −44.57 (*s*). HRMS (*m*/*z* calculated for C_34_H_35_Fe_2_NaO_4_P) [*M* + Na]^+^ 673.0869; found 673.0877.

#### Synthesis of mono­vinyl monoacetyl phosphine sulfide 8a, mono­vinyl mono­hydroxy phosphine sulfide 8b and monohy­droxy mono­acetyl phosphine sulfide 8c   

Di­amine phosphine sulfide **3** (653 mg, 1.00 mmol; Honegger & Widhalm, 2019*a* ▸) was suspended in Ac_2_O (1 ml) in a flame-dried Schlenk tube. The suspension was degassed and stirred under argon for 7 d at room temperature, then for 7 h at 100 °C until the starting material was com­pletely consumed (thin-layer chromatography, TLC). Ac_2_O was removed under reduced pressure and the residue was purified by column chromatography (SiO_2_, 0–100% EtOAc in hepta­ne), yielding several mixed fractions, as well as com­pounds **8a** (yield 135 mg, 22%), **8b** (yield 99 mg, 17%) and **8c** (yield 74 mg, 12%) as orange crystals upon removal of the solvent.

Analytical data for **8a**: m.p. 177–178 °C. ^1^H NMR (600 MHz, CDCl_3_): δ 8.13 (*dd*, *J* = 17.7, 10.8 Hz, 1H), 7.77 (*dd*, *J* = 13.3, 7.4 Hz, 2H), 7.47–7.41 (*m*, 3H), 6.49 (*q*, *J* = 6.4 Hz, 1H), 5.46 (*dd*, *J* = 17.7, 1.7 Hz, 1H), 5.16 (*dd*, *J* = 10.8, 1.6 Hz, 1H), 4.84 (*m*, 1H), 4.59 (*m*, 1H), 4.36 (*s*, 5H), 4.30 (*m*, 1H), 4.28 (*m*, 1H), 4.14 (*s*, 5H), 3.78 (*m*, 1H), 3.54 (*m*, 1H), 1.57 (*d*, *J* = 6.5 Hz, 3H), 1.04 (*s*, 3H). ^13^C{^1^H} NMR: δ 169.39 (C_q_), 135.44 (*d*, *J*_CP_ = 88.3 Hz, C_q_), 134.47 (CH), 132.29 (*d*, *J*_CP_ = 10.5 Hz, CH), 130.77 (*d*, *J*_CP_ = 2.8 Hz, CH), 127.49 (*d*, *J*_CP_ = 12.2 Hz, CH), 111.38 (CH_2_), 88.95 (*d*, *J*_CP_ = 12.1 Hz, C_q_), 88.43 (*d*, *J*_CP_ = 12.0 Hz, C_q_), 79.19 (*d*, *J*_CP_ = 95.2 Hz, C_q_), 75.84 (*d*, *J*_CP_ = 11.4 Hz, CH), 75.03 (*d*, *J*_CP_ = 12.0 Hz, CH), 74.46 (*d*, *J*_CP_ = 95.1 Hz, C_q_), 71.15 (CH), 70.94 (CH), 70.60 (*d*, *J*_CP_ = 9.0 Hz, CH), 69.88 (*d*, *J*_CP_ = 10.2 Hz, CH), 68.40 (*d*, *J*_CP_ = 10.3 Hz, CH), 68.14 (*d*, *J*_CP_ = 8.9 Hz, CH), 67.99 (CH), 20.03 (CH_3_), 18.49 (CH_3_). ^31^P NMR: δ 39.14 (*s*). HRMS (*m*/*z* calculated for C_32_H_31_Fe_2_O_2_PS) [*M*]^+^ 622.0481, found 622.0462; [*M* + Na]^+^ 645.0379, found 645.0358; [*M* + K]^+^ 661.0118, found 661.0104.

Analytical data for **8b**: m.p. 205–206 °C (decom­position). ^1^H NMR (600 MHz, CDCl_3_): δ 8.10 (*dd*, *J* = 17.6, 10.8 Hz, 1H), 7.87–7.81 (*m*, 2H), 7.51–7.42 (*m*, 3H), 5.49 (*dd*, *J* = 17.6, 1.6 Hz, 1H), 5.23–5.17 (*m*, 1H), 5.20 (*dd*, *J* = 10.8, 1.7 Hz, 1H), 4.88 (*m*, 1H), 4.49 (*m*, 1H), 4.34 (*s*, 5H), 4.33 (*m*, 1H), 4.24 (*m*, 1H), 4.17 (*s*, 5H), 3.77 (*m*, 1H), 3.71 (*m*, 1H), 2.41 (*d*, *J* = 5.3 Hz, 1H), 1.26 (*d*, *J* = 6.6 Hz, 3H). ^13^C{^1^H} NMR: δ 135.30 (*d*, *J*_CP_ = 87.1 Hz, C_q_), 134.25 (CH), 132.10 (*d*, *J*_CP_ = 10.3 Hz, CH), 131.38 (*d*, *J*_CP_ = 2.8 Hz, CH), 127.96 (*d*, *J*_CP_ = 12.1 Hz, CH), 111.74 (CH_2_), 94.90 (*d*, *J*_CP_ = 12.3 Hz, C_q_), 88.43 (*d*, *J*_CP_ = 11.8 Hz, C_q_), 78.56 (*d*, *J*_CP_ = 95.4 Hz, C_q_), 75.05 (*d*, *J*_CP_ = 12.0 Hz, CH), 74.97 (*d*, *J*_CP_ = 12.6 Hz, CH), 73.48 (*d*, *J*_CP_ = 96.0 Hz, C_q_), 71.22 (CH), 71.00 (*d*, *J*_CP_ = 9.7 Hz, CH), 70.72 (CH), 70.10 (*d*, *J*_CP_ = 10.4 Hz, CH), 68.38 (*d*, *J*_CP_ = 9.1 Hz, CH), 68.04 (*d*, *J*_CP_ = 10.5 Hz, CH), 64.38 (CH), 21.91 (CH_3_). ^31^P NMR: δ 40.52 (*s*). HRMS (*m*/*z* calculated for C_30_H_29_Fe_2_OPS) [*M*]^+^ 580.0376, found 580.0360; [*M* + Na]^+^ 603.0273, found 603.0273; [*M* + K]^+^ 619.0013, found 619.0018.

Analytical data for **8c**: m.p. 174–175 °C. ^1^H NMR (600 MHz, CDCl_3_): δ 8.20–8.14 (*m*, 2H), 7.57–7.54 (*m*, 3H), 6.60 (*q*, *J* = 6.3 Hz, 1H), 4.93 (*m*, 1H), 4.67 (*m*, 2H), 4.49 (*m*, 1H), 4.38 (*m*, 1H), 4.34 (*m*, 1H), 4.11 (*s*, 5H), 4.10 (*s*, 5H), 1.90 (*s*, 3H), 1.83 (*d*, *J* = 6.3 Hz, 3H), 1.63 (*m*, 1H), 1.43 (*dd*, *J* = 19.2, 6.4 Hz, 1H), 1.40 (*d*, *J* = 6.6 Hz, 3H). ^13^C{^1^H} NMR: δ 169.70 (C_q_), 135.42 (*d*, *J*_CP_ = 87.3 Hz, C_q_), 132.21 (*d*, *J*_CP_ = 10.5 Hz, CH), 131.47 (*d*, *J*_CP_ = 2.9 Hz, CH), 127.88 (*d*, *J*_CP_ = 12.2 Hz, CH), 93.49 (*d*, *J*_CP_ = 11.8 Hz, C_q_), 93.47 (*d*, *J*_CP_ = 13.5 Hz, C_q_), 75.85 (*d*, *J*_CP_ = 13.4 Hz, CH), 74.53 (C_q_), 73.91 (C_q_), 71.53 (*d*, *J*_CP_ = 9.3 Hz, CH), 70.93 (CH), 70.60 (*d*, *J*_CP_ = 60.4 Hz, CH), 70.58 (CH), 70.27 (*d*, *J*_CP_ = 9.3 Hz, CH), 69.48 (*d*, *J*_CP_ = 10.1 Hz, CH), 68.87 (CH), 68.56 (*d*, *J*_CP_ = 10.9 Hz, CH), 64.20 (CH), 22.37 (CH_3_), 22.23 (CH_3_), 21.80 (CH_3_). ^31^P NMR: δ 39.12 (*s*). HRMS (*m*/*z* calculated for C_32_H_33_Fe_2_O_3_PS) [*M*]^+^ 640.0587, found 640.0566; [*M* + Na]^+^ 663.0485, found 663.0463.

#### Catalytic experiments: asymmetric allylic alkyl­ation (Widhalm *et al.*, 1996 ▸)   

In a flame-dried Schlenk tube, the diferrocene ligand (0.010 mmol, 1 mol%) and [Pd(all­yl)Cl]_2_ (1.8 mg, 0.005 mmol, 0.5 mol%) were dissolved in degassed DCM (1 ml) in that order under argon. The yellow solution was stirred for 20 min while it turned orange. To the solution, freshly distilled 1,3-di­phenyl­allyl acetate (252 mg, 1.00 mmol), dimethyl malonate (0.340 ml, 3.00 mmol, 3 equiv.), bis­(tri­methyl­sil­yl)acetamide (0.740 ml, 3.00 mmol, 3 equiv.) and a catalytic amount of potassium acetate were added in that order. The reaction mixture was degassed once and stirred for 48 h at room temperature until the catalytic conversion was com­plete. To the solution, Et_2_O (15 ml) was added. The organic layer was washed twice with saturated aqueous NH_4_Cl solution, dried over Na_2_SO_4_ and the solvent removed under reduced pressure. The residue was dried, dissolved in DCM (2 ml) and filtered through SiO_2_. The enanti­omeric excess (e.e.) was detected *via* chiral high-performance liquid chromatography (HPLC; Chiralcel OD-H, 2% iso­propanol in *n*-hepta­ne).

### Melting points   

The melting points were measured on a Reichelt Thermovar Kofler apparatus and are uncorrected.

### Chiral high-performance liquid chromatography (HPLC)   

HPLC analysis was performed on an Agilent Technologies 1200 series system using a Chiralcel OD-H chiral column.

### NMR spectroscopy   

Routine NMR spectra were recorded on a 400 MHz Bruker AVIII 400 spectrometer operating at 400.27 (^1^H), 100.66 (^13^C) and 162.04 MHz (^31^P) with an autosampler. The ^1^H and ^13^C{^1^H} NMR spectra used for substance characterization were recorded either on a 600 MHz Bruker AVIII 600 spectrometer operating at 600.25 (^1^H) and 150.95 MHz (^13^C) or on a Bruker AVIII 700 spectrometer operating at 700.40 (^1^H) and 176.13 MHz (^13^C). ^13^C NMR spectra were recorded in J-modulated mode. NMR chemical shifts are referenced to nondeuterated CHCl_3_ residual shifts at 7.26 ppm for ^1^H NMR and to CDCl_3_ at 77.00 ppm for ^13^C NMR. Coupling patterns in the ^1^H and ^13^C NMR spectra are denoted using standard abbreviations: *s* (singlet), *d* (doublet), *t* (triplet), *q* (quartet), *m* (multiplet) and *p* (pseudo). For the ^13^C NMR spectra, carbon resonances were identified as C_q_, CH, CH_2_ and CH_3_.

### High-resolution mass spectroscopy (HRMS)   

HRMS were recorded by a Bruker Maxis ESI oa-RTOF mass spectrometer equipped with a quadrupole analyzer ion guide.

### Preparative column chromatography   

Preparative column chromatography was carried out on an Biotage Isolera One automated flash chromatography instrument using self-packed columns containing either SiO_2_ (Macherey–Nagel silica gel 60M, particle size 40–63 µm) or Al_2_O_3_ (Merck aluminium oxide 90 standardized, activation grade II–III).

### X-ray diffractometry   

X-ray diffraction was performed on a Bruker X8 APEXII diffractometer, a Bruker D8 Venture diffractometer and a Bruker APEXII CCD diffractometer, all with Mo *K*α radiation.

### Refinement   

The structures were solved by direct methods and refined using full-matrix least-squares techniques. Non-H atoms were refined with anisotropic displacement parameters. H atoms were inserted at calculated positions and refined using a riding model. C—H bond lengths in the aromatic and olefin bond systems were constrained at 0.950 Å, aliphatic CH_2_ groups at 0.990 Å and aliphatic CH_3_ groups at 0.980 Å. The default values of *SHELXL* (Sheldrick, 2008 ▸) were used for the riding-atom model. Fixed *U*_iso_ values of 1.2 times were used for all C(H) and C(H,H) groups, and fixed *U*_iso_ values of 1.5 times were used for all C(H,H,H) and O(H) groups. Details for each com­pound are summarized in the CIF file under the keyword ‘_refine_special_details’.

The position of the acidic atom H1*B* at **8b** was stabilized using a length-fixing restraint. Several reflections, primarily inner ones, have been omitted to avoid wrong interpretations.

The amine H atom of com­pound **9b** was refined taking account of the two possible configurations of the N atom. The choice was stable and in agreement with the position of available electron density.

Crystal data, data collection and structure refinement details are summarized in Table 1 ▸.

## Results and discussion   

**Figure 2:**
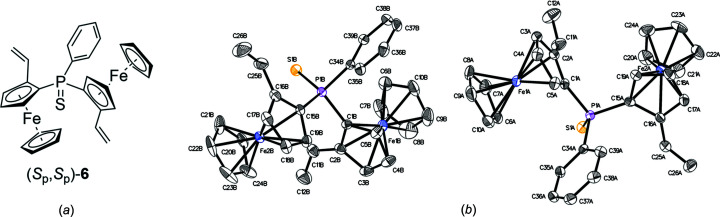
(*a*) Chemical structure and (*b*) displacement ellipsoid plot of divinyl **6**. The ellipsoid probability level of this figure and all subsequent figures is 50%.

The syntheses carried out in the framework of this study are summarized in Fig. 1 ▸. The structure of the central diferrocene precursor **2** has been deposited previously (Steiner & Pioda, 1999 ▸) in the Cambridge Structural Database (Groom *et al.*, 2016 ▸). First, we eliminated the di­methyl­amine groups of **2** to obtain divinyl structure (*S*_p_,*S*_p_)-**5** by heating in acetic anhydride according to Honegger & Widhalm (2020 ▸). The sensitive phosphine was then protected by reaction with elemental sulfur to qu­anti­tatively produce di­vinyl­phosphine sulfide (*S*_p_,*S*_p_)-**6**, shown in Fig. 2 ▸ (Honegger *et al.*, 2020 ▸). The substance was readily isolated as orange crystals upon removal of the solvent. Cyclization attempts of **6** *via* ring-closing metathesis (RCM) failed, possibly due to the separation of the vinyl C atoms, steric strain in the product or inter­ference of the Grubbs catalyst with the phosphine sulfide. However, Lewis acid-catalyzed hydro­vinyl­ation afforded the desired all-carbon backbone (Honegger & Widhalm, 2020 ▸).

**Figure 3:**
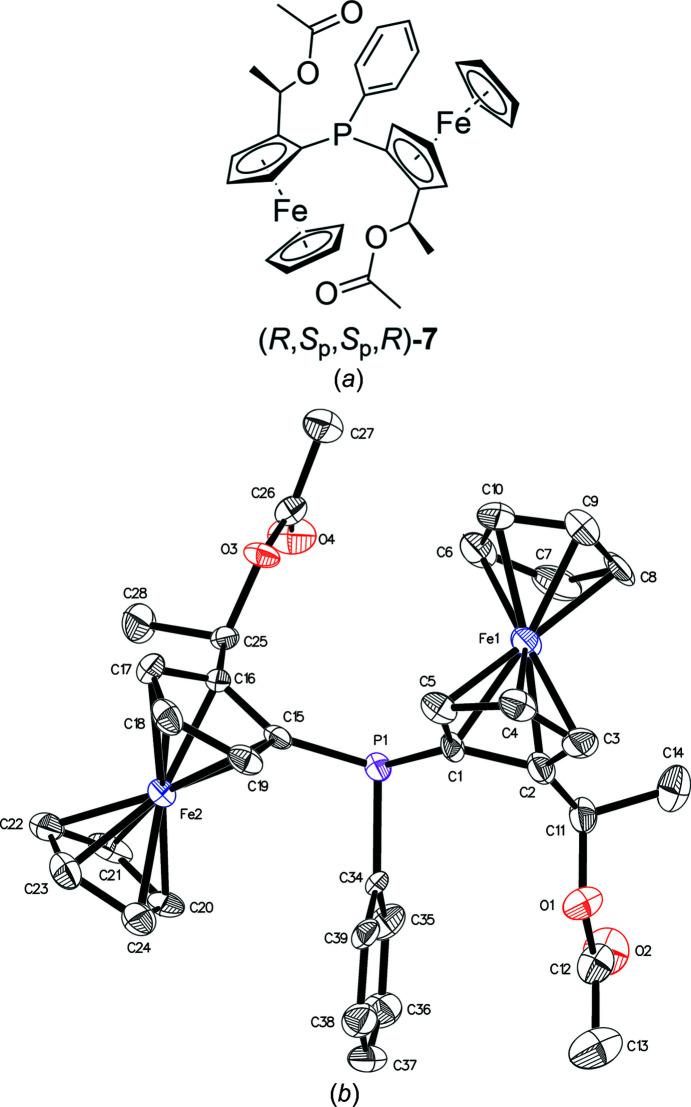
(*a*) Chemical structure and (*b*) displacement ellipsoid plot of di­acetate **7**.

**Figure 4:**
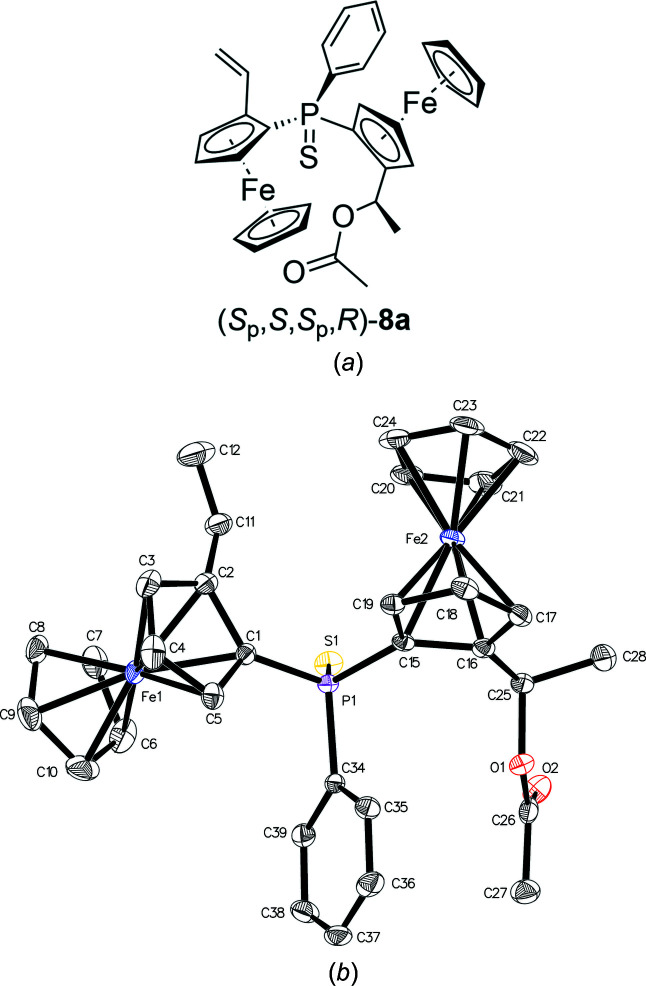
(*a*) Chemical structure and (*b*) displacement ellipsoid plot of mono­acetate **8a**.

**Figure 5:**
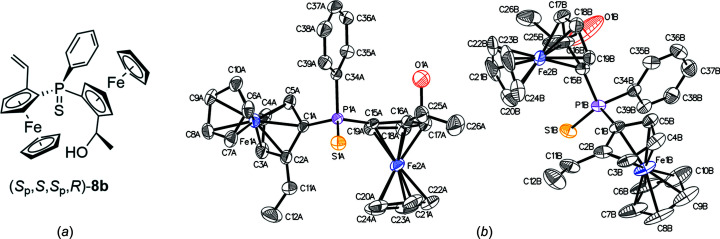
(*a*) Chemical structure and (*b*) displacement ellipsoid plot of mono­hydroxide **8b**.

**Figure 6:**
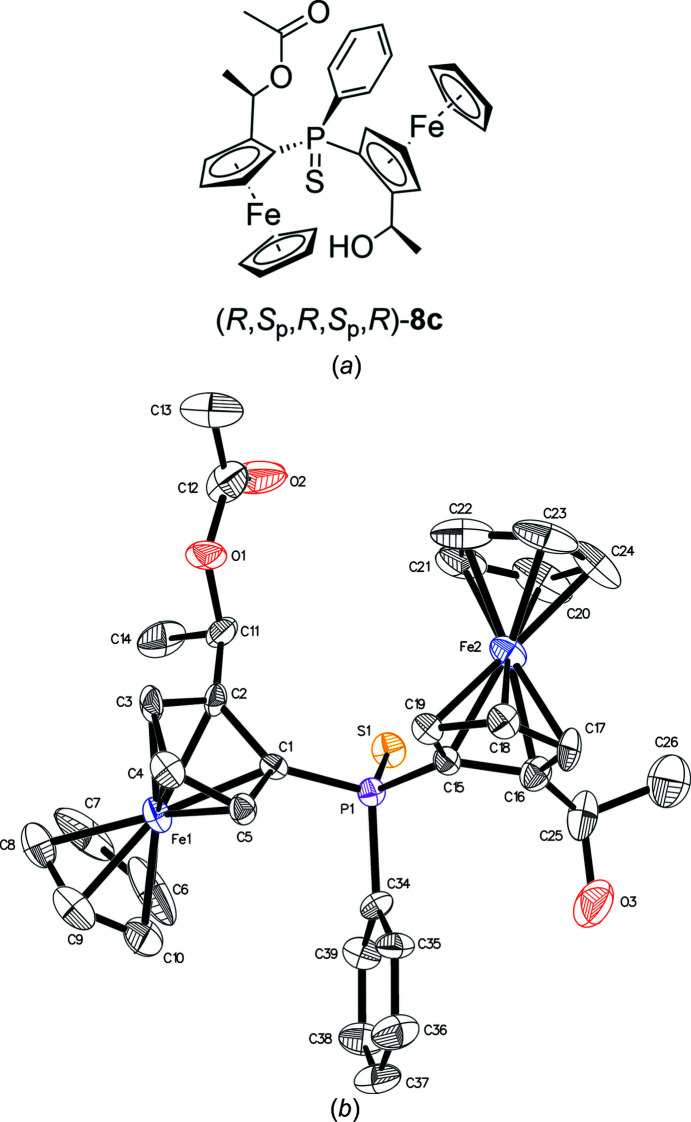
(*a*) Chemical structure and (*b*) displacement ellipsoid plot of mono­acetate­mono­hydroxide **8c**.

For an alternative approach, we replaced the di­amino groups with more capable leaving groups in order to close the ring with bidentate nucleophiles. In one of these attempts, we replaced the amines by acetate groups using acetic anhydride. The resulting di­acetate (*R*,*S*_p_,*S*_p_,*R*)-**7** crystallized upon removal of the solvent (Fig. 3 ▸). Alternatively, the reactive phosphino group was protected by reaction with elemental sulfur to yield phosphine sulfide (*R*,*S*_p_,*S*_p_,*R*)-**3** (Honegger & Widhalm, 2019*a* ▸). In contrast to the unprotected phosphine, we could not obtain the di­acetate from derivative (*R*,*S*_p_,*S*_p_,*R*)-**3**, but from the reaction mixture, three com­pounds, namely, (*S*_p_,*S*,*S*_p_,*R*)-**8a** (Fig. 4 ▸), (*S*_p_,*S*,*S*_p_,*R*)-**8b** (Fig. 5 ▸) and (*R*,*S*_p_,*R*,*S*_p_,*R*)-**8c** (Fig. 6 ▸) with acetate, hy­droxy or vinyl side groups instead, could be isolated, indicating that the substitution was followed by elimination or cleavage of the acetyl group.

**Figure 7:**
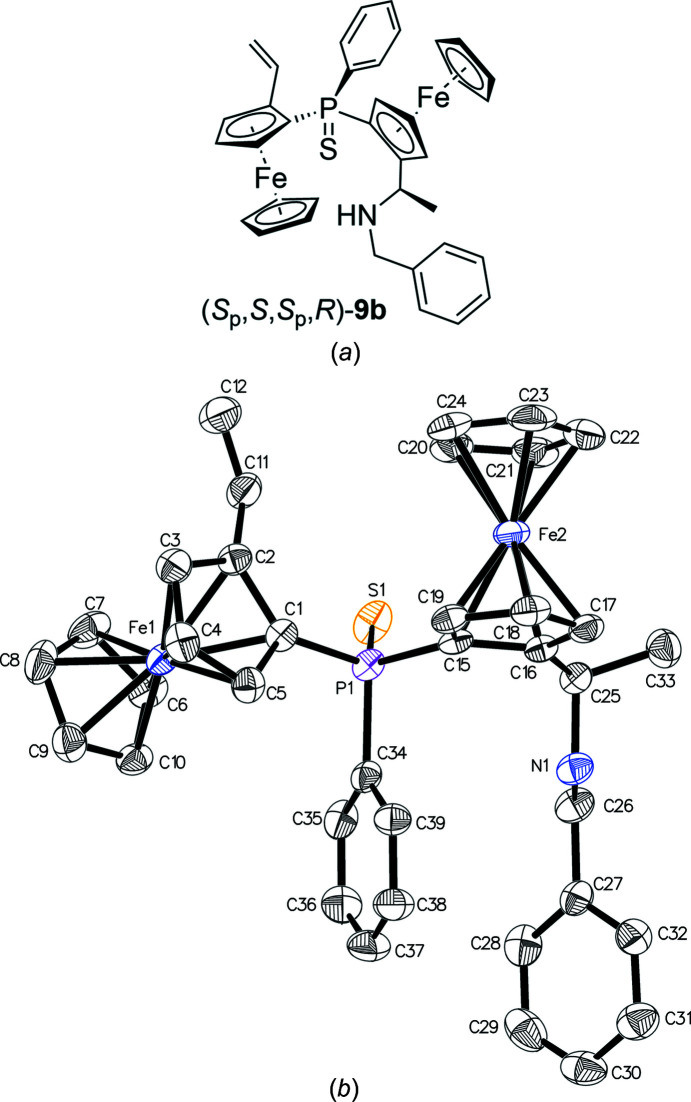
(*a*) Chemical structure and (*b*) displacement ellipsoid plot of benzyl­amine **9b**.

Alternatively, an attempt was made to convert the di­amine (*R*,*S*_p_,*S*_p_,*R*)-**3** into a di­ammonium salt, yet only monome­thio­dide **4** was obtained in qu­anti­tative yield. Bridging with benzyl­amine afforded ring-closed **9a** along with the mono-eliminated crystalline side product (*S*_p_,*S*,*S*_p_,*R*)-**9b** (Fig. 7 ▸), both in poor yield (12 and 13%, respectively) (Honegger & Widhalm, 2019*b* ▸).

**Figure 8:**
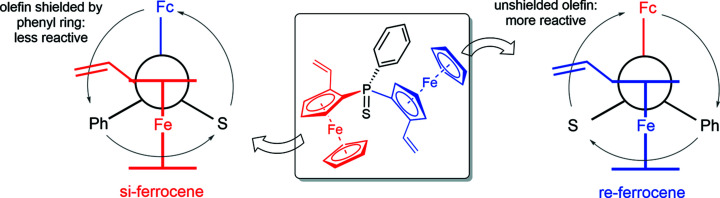
The *re*/*si* nomenclature for diferrocenes developed in the framework of this study to distinguish between the two ferrocenyl subunits.

**Figure 9:**
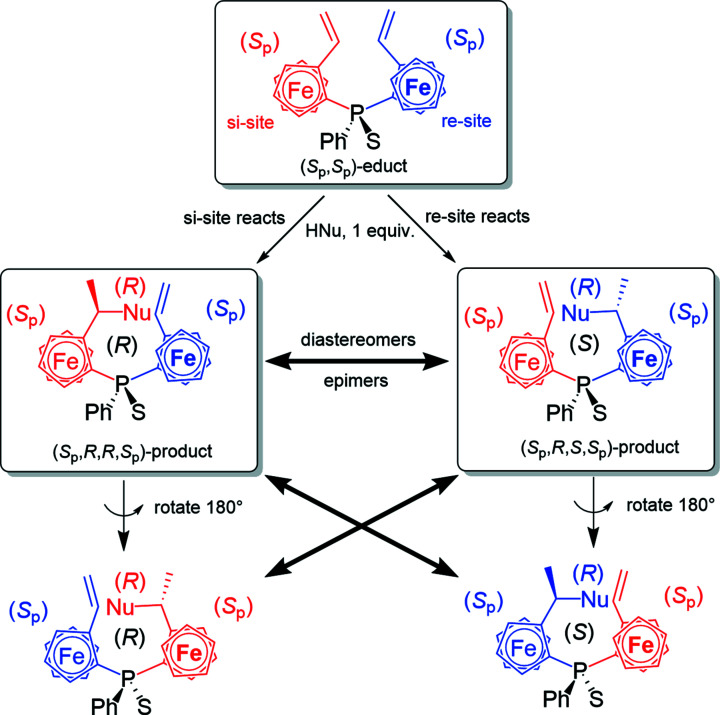
Hypothetical Markovnikov-selective addition of HNu to a symmetrical di(vinyl­ferrocene), with Nu standing for an arbitrary nucleophile. The other Cp rings have been omitted for clarity and represented indirectly by the planar–chiral configuration (*S*_p_).

In the symmetric diferrocenes (*S*_p_,*S*_p_)-**6** and (*S*_p_,*S*_p_)-**7**, the ferrocene subunits are identical. Fig. 8 ▸ shows a system developed to distinguish them into an *re*-site and an *si*-site. Taking divinyl com­pound (*S*_p_,*S*_p_)-**6** as an example, both ferrocene units are planar–chiral (*S*_p_). The P atom in com­pound (*S*_p_,*S*_p_)-**6** is prochiral; if any of the two vinyl­ferrocene groups are modified, the ferrocene substituents become distinguishable and the P atom thus a chiral centre. Fig. 9 ▸ shows a hypothetical Markovnikov regioselective addition of HNu to divinyl (*S*_p_,*S*_p_)-**6**, Nu standing for a generalized nucleophile. De­pen­ding on whether HNu is added to the *re*-site or *si*-site vinyl group, the P atom becomes an (*S*)- or (*R*)-chiral centre, respectively. The two possible products are diastereomers since the reaction turns the P-atom centre from prochiral to centre-chiral. In addition, this reaction introduces a new chiral centre, but in a diastereoselective fashion since one of the two sites is blocked by the other ring of the ferrocenyl unit (Marquarding *et al.*, 1970 ▸). The two possible products are diastereomers, differing only in the resulting configuration of the P atom (epimers). In the com­pounds presented in this article, we know the configuration at the (*S*_p_)-disubstituted ferrocene will be selectively (*R*), since the approach of the nucleophile from the (*S*)-site is blocked by the other cyclo­penta­dienyl (Cp) ring (Marquarding *et al.*, 1970 ▸). Fig. 9 ▸ illustrates this by rotating the two possible products by 180° for better com­parison with the other product; again, only the configuration of the P atom differs. We only observed the formation of one of the two possible diastereomers, hence the reaction proceeds diastereoselectively, as was observed for different reactions throughout this study. Typically, the *re*-site of the symmetrical (*S*_p_,*S*_p_)-precursors was more reactive.

Thus, the vinyl­ferrocene groups are diastereotopic and their respective NMR shifts can be distinguished despite their apparent equality in connectivity when neglecting stereochemical aspects. In fact, protons attached to the inner vinyl protons in (*S*_p_,*S*_p_)-**6** differ so greatly in chemical shift that one of the two is found at a higher field than even aromatic protons (δ = 8.1 ppm; Honegger *et al.*, 2020 ▸).

The preferred conformation of ferrocenyl units with both Cp rings is a perpendicular arrangement, with the reactive *re*-ferrocene closer to the small sulfur residue and the less reactive *si*-ferrocene shielded by the bulkier phenyl group.

For the asymmetric diferrocene com­pounds (*S*_p_,*S*,*S*_p_,*R*)-**8a**, (*S*_p_,*S*,*S*_p_,*R*)-**8b** and (*S*_p_,*S*,*S*_p_,*R*)-**9b**, the ferrocenyl at the smaller sulfur group bears the more bulky substituent (acetate, hy­droxy and benz­yl), while the other ferrocenyl unit at the larger phenyl ring is substituted with a sterically less demanding vinyl group. Only hy­droxy­acetate (*S*_p_,*R*,*S*_p_,*R*)-**8c** shows the opposite preference. The preferred geometry might be mainly controlled by subtle inter- and intra­molecular steric inter­actions as no π–π inter­actions could be detected. The protic H atoms in com­pounds **8b**, **8c** (O—H group) and **9b** (N—H group) form intra­molecular hydrogen bonds with the π-system of the P-substituted phenyl group.

Since we obtained the sufficiently stable com­pound (*S*_p_,*S*_p_)-**6** in large enough qu­anti­ties, we tested its asymmetry-inducing performance as a ligand in an *in-situ* formed Pd^II^ com­plex used for asymmetric allylic alkyl­ation according to Widhalm *et al.* (1996 ▸). This purely planar–chiral com­pound achieved an enanti­omeric excess of 35%, which is less than what we found for previously known (*R*,*S*_p_,*S*_p_,*R*)-**2** (57%). Regardless, the coordination structure of (*S*_p_,*S*_p_)-**6** and Pd^II^ remains an inter­esting question since neither phosphine (*S*_p_,*S*_p_)-**5** nor its phosphine oxide analog were able to activate Pd^II^. We could not isolate the catalytically active Pd^II^ com­plex to study its structure, but we speculate that the phosphine sulfide might act as an electron donor to form a dative bond in transition-metal catalysts.

## Conclusion   

We present the crystal structures of six homochiral phospho­rous-linked diferrocenes. All the ferrocene units are planar–chiral (*S*_p_) and five of the com­pounds include one or two centre-chiral C atoms (*R*) also. Inter­estingly, the mol­ecules lack strong inter­molecular inter­actions and exhibit no π-stacking, even though most of the C atoms are aromatic. Compounds **8b**, **8c** and **9b** include acidic H atoms (*R*O—H and *R*_2_N—H) capable of forming hydrogen bridges with the π-electron systems of the phenyl ring.

Four of the presented com­pounds contain two differently substituted ferrocene units, [(Fc^A^)(Fc^B^)(Ph)P], while in the other two com­pounds, the two ferrocene com­pounds are equal, [(Fc^A^)_2_(Ph)P]. Due to the planar chirality of the ferrocene units, the linking P atom is prochiral and one of the two equal ferrocene units reacts far more readily with reagents than the other due to diastereoselectivity. This ability of selective chemical ferrocene subunit differentiation suggests the application of such diferrocenes in asymmetric organic chemistry, for instance, as ligands, catalysts and auxiliaries.

## Supplementary Material

Crystal structure: contains datablock(s) global, 7, 8a, 8b, 8c, 9b, 6. DOI: 10.1107/S2053229621001996/zo3008sup1.cif

Structure factors: contains datablock(s) 6. DOI: 10.1107/S2053229621001996/zo30086sup2.hkl

Supporting information file. DOI: 10.1107/S2053229621001996/zo30086sup8.mol

Structure factors: contains datablock(s) 7. DOI: 10.1107/S2053229621001996/zo30087sup3.hkl

Supporting information file. DOI: 10.1107/S2053229621001996/zo30087sup9.mol

Supporting information file. DOI: 10.1107/S2053229621001996/zo30088asup10.mol

Structure factors: contains datablock(s) 8a. DOI: 10.1107/S2053229621001996/zo30088asup4.hkl

Supporting information file. DOI: 10.1107/S2053229621001996/zo30088bsup11.mol

Structure factors: contains datablock(s) 8b. DOI: 10.1107/S2053229621001996/zo30088bsup5.hkl

Supporting information file. DOI: 10.1107/S2053229621001996/zo30088csup12.mol

Structure factors: contains datablock(s) 8c. DOI: 10.1107/S2053229621001996/zo30088csup6.hkl

Supporting information file. DOI: 10.1107/S2053229621001996/zo30089bsup13.mol

Structure factors: contains datablock(s) 9b. DOI: 10.1107/S2053229621001996/zo30089bsup7.hkl

CCDC references: 1960106, 1960104, 1960103, 1960105, 1943185, 1943184

## Figures and Tables

**Table:** 

	**6**	**7**	**8a**
Crystal data			
Chemical formula	[Fe_2_(C_5_H_5_)_2_(C_20_H_17_PS)]	[Fe_2_(C_5_H_5_)_2_(C_24_H_25_O_4_P)]	[Fe_2_(C_5_H_5_)_2_(C_22_H_21_O_2_PS)]
*M*_r_	562.24	650.29	622.30
Crystal system, space group	Monoclinic, *P*2_1_	Orthorhombic, *P*2_1_2_1_2_1_	Orthorhombic, *P*2_1_2_1_2_1_
Temperature (K)	130	130	100
*a*, *b*, *c* (Å)	8.4709 (7), 14.1401 (11), 21.0310 (17)	7.631 (2), 10.877 (2), 36.025 (8)	7.4923 (3), 12.0133 (4), 31.758 (1)
α, β, γ (°)	90, 91.880 (3), 90	90, 90, 90	90, 90, 90
*V* (Å^3^)	2517.7 (4)	2990.2 (12)	2858.45 (17)
*Z*	4	4	4
Radiation type	Mo *K*α	Mo *K*α	Mo *K*α
μ (mm^−1^)	1.32	1.06	1.17
Crystal size (mm)	0.28 × 0.25 × 0.13	0.15 × 0.08 × 0.06	0.21 × 0.14 × 0.05

Data collection			
Diffractometer	Bruker X8 APEXII	Bruker X8 APEXII	Bruker D8 Venture
Absorption correction	Multi-scan (*SADABS*; Bruker, 2008 ▸	Multi-scan (*SADABS*; Bruker, 2008 ▸)	Multi-scan (*SADABS*; Bruker, 2012 ▸)
*T*_min_, *T*_max_	0.620, 0.746	0.562, 0.745	0.607, 0.746
No. of measured, independent and observed [*I* > 2σ(*I*)] reflections	35484, 14468, 13352	46099, 5441, 4085	52739, 8330, 7214
*R*_int_	0.038	0.150	0.062
(sin θ/λ)_max_ (Å^−1^)	0.708	0.602	0.704

Refinement			
*R*[*F* ^2^ > 2σ(*F* ^2^)], *wR*(*F* ^2^), *S*	0.032, 0.071, 1.03	0.065, 0.122, 1.04	0.032, 0.062, 1.04
No. of reflections	14468	5441	8330
No. of parameters	613	374	345
No. of restraints	1	0	0
H-atom treatment	H-atom parameters constrained	H-atom parameters constrained	H-atom parameters constrained
Δρ_max_, Δρ_min_ (e Å^−3^)	0.42, −0.37	0.94, −0.43	0.34, −0.34
Absolute structure	Flack *x* determined using 5751 quotients [(*I* ^+^) − (*I* ^−^)]/[(*I* ^+^) + (*I* ^−^)] (Parsons *et al.*, 2013 ▸)	Flack *x* determined using 1235 quotients [(*I* ^+^) − (*I* ^−^)]/[(*I* ^+^) + (*I* ^−^)] (Parsons *et al.*, 2013 ▸)	Flack *x* determined using 2809 quotients [(*I* ^+^) − (*I* ^−^)]/[(*I* ^+^) + (*I* ^−^)] (Parsons *et al.*, 2013 ▸)
Absolute structure parameter	−0.019 (5)	−0.02 (2)	−0.007 (5)

**Table:** 

	**8b**	**8c**	**9b**
Crystal data			
Chemical formula	[Fe_2_(C_5_H_5_)_2_(C_20_H_19_OPS)]	[Fe_2_(C_5_H_5_)_2_(C_22_H_23_O_3_PS)]	[Fe_2_(C_5_H_5_)_2_(C_27_H_26_NPS)]
*M*_r_	580.26	640.31	669.40
Crystal system, space group	Orthorhombic, *P*2_1_2_1_2_1_	Orthorhombic, *P*2_1_2_1_2_1_	Orthorhombic, *P*2_1_2_1_2_1_
Temperature (K)	130	130	130
*a*, *b*, *c* (Å)	7.5285 (3), 17.6463 (7), 39.3333 (15)	7.8204 (11), 17.835 (3), 20.394 (2)	12.3578 (9), 14.4342 (10), 17.4796 (15)
α, β, γ (°)	90, 90, 90	90, 90, 90	90, 90, 90
*V* (Å^3^)	5225.4 (4)	2844.5 (7)	3117.9 (4)
*Z*	8	4	4
Radiation type	Mo *K*α	Mo *K*α	Mo *K*α
μ (mm^−1^)	1.27	1.18	1.08
Crystal size (mm)	0.25 × 0.2 × 0.17	0.1 × 0.06 × 0.01	0.22 × 0.11 × 0.09

Data collection			
Diffractometer	Bruker X8 APEXII	Bruker X8 APEXII	Bruker APEXII CCD
Absorption correction	Multi-scan (*SADABS*; Bruker, 2008 ▸)	Multi-scan (*SADABS*; Bruker, 2008 ▸)	Multi-scan (*SADABS*; Bruker, 2008 ▸)
*T*_min_, *T*_max_	0.650, 0.746	0.568, 0.745	0.486, 0.746
No. of measured, independent and observed [*I* > 2σ(*I*)] reflections	38763, 14944, 11780	35430, 5258, 3417	38997, 9160, 7062
*R*_int_	0.045	0.167	0.075
(sin θ/λ)_max_ (Å^−1^)	0.704	0.606	0.706

Refinement			
*R*[*F* ^2^ > 2σ(*F* ^2^)], *wR*(*F* ^2^), *S*	0.056, 0.137, 1.04	0.054, 0.093, 1.02	0.040, 0.091, 0.98
No. of reflections	14944	5258	9160
No. of parameters	635	356	384
No. of restraints	1	0	0
H-atom treatment	H-atom parameters constrained	H-atom parameters constrained	H atoms treated by a mixture of independent and constrained refinement
Δρ_max_, Δρ_min_ (e Å^−3^)	2.08, −1.80	0.42, −0.41	0.43, −0.35
Absolute structure	Flack *x* determined using 4140 quotients [(*I* ^+^) − (*I* ^−^)]/[(*I* ^+^) + (*I* ^−^)] (Parsons *et al.*, 2013 ▸)	Flack *x* determined using 1051 quotients [(*I* ^+^) − (*I* ^−^)]/[(*I* ^+^) + (*I* ^−^)] (Parsons *et al.*, 2013 ▸)	Flack *x* determined using 2549 quotients [(*I* ^+^) − (*I* ^−^)]/[(*I* ^+^) + (*I* ^−^)] (Parsons *et al.*, 2013 ▸)
Absolute structure parameter	−0.010 (7)	0.00 (2)	−0.016 (10)

Computer programs: *APEX2* (Bruker, 2009 ▸), *APEX3* (Bruker, 2016 ▸), *SAINT* (Bruker, 2009 ▸, 2016 ▸), *SHELXS97* (Sheldrick, 2008 ▸), *SHELXL97* (Sheldrick, 2008 ▸) and *OLEX2* (Dolomanov *et al.*, 2009 ▸).
